# Evaluation of Antiepileptic Drugs’ Stability in Oral Fluid Samples

**DOI:** 10.3390/ph18071049

**Published:** 2025-07-17

**Authors:** João Martinho, Ana Y. Simão, Tiago Rosado, Eugenia Gallardo

**Affiliations:** 1 RISE-Health, Departamento de Ciências Médicas, Faculdade de Ciências da Saúde, Universidade da Beira Interior, Av. Infante D. Henrique, 6200-506 Covilhã, Portugal; joao.pedro.martinho@ubi.pt (J.M.); anaaysa95@gmail.com (A.Y.S.); tiago.rosado@ubi.pt (T.R.); 2 Laboratório de Fármaco-Toxicologia-UBIMedical, Estrada Municipal 506, 6200-284 Covilhã, Portugal; 3 Centro Académico Clínico das Beiras (CACB), Grupo de Problemas Relacionados com Toxicofilias, UBIMedical, Universidade da Beira Interior, Estrada Municipal 506, 6200-284 Covilhã, Portugal

**Keywords:** antiepileptic drugs, oral fluid, stability, HPLC-DAD

## Abstract

**Background/Objectives:** Epilepsy affects approximately 50 million people worldwide, with antiepileptic drugs (AEDs) remaining the cornerstone of treatment. Due to their narrow therapeutic windows, AEDs are ideal candidates for therapeutic drug monitoring (TDM). Oral fluid is increasingly considered a viable alternative to blood and urine, as it reflects the free (active) concentration of many AEDs. Its non-invasive collection, which does not require trained personnel, makes it particularly suitable for TDM in paediatric and geriatric populations. However, as samples are often stored for extended periods before analysis, analyte stability becomes a critical concern. This study aimed to evaluate the stability of four commonly used AEDs in dried saliva spot (DSS) samples. **Methods:** Phenobarbital, phenytoin, carbamazepine, and carbamazepine-10,11-epoxide were analysed in oral fluid samples collected via spitting and stored as DSSs. Quantification was performed using high-performance liquid chromatography with diode array detection (HPLC-DAD). Design of experiments tools were used to assess the effects of preservatives, storage temperatures, light exposure, and storage durations on analyte stability. **Results:** Optimal conditions were refrigeration in the dark, with a low concentration of ascorbic acid as preservative. Samples at 10 µg/mL remained stable for 14 days longer than those without preservative or reported in previous studies. Unexpectedly, at 0.5 µg/mL, analytes in samples without preservative showed greater stability. **Conclusions:** To our knowledge, this is the first study combining DSS and HPLC-DAD to assess the stability of these AEDs in oral fluid, providing valuable insights for non-invasive TDM strategies and supporting the feasibility of saliva-based monitoring in clinical settings.

## 1. Introduction

Epilepsy affects around 50 million people worldwide and is considered a highly heterogeneous condition, with an annual incidence ranging from 50.4 to 81.7 cases per 100,000 people. It presents a wide range of causes, outcomes, and treatment options [1,2]. The regular manifestation of unprovoked seizures is the actual definition of epilepsy, which means that, despite the fact that everyone has a 10% lifetime risk of experiencing a single epileptic seizure, the majority of those who do so are not really diagnosed with this condition [1,3].

The primary treatment for patients with epilepsy is the employment of antiepileptic drugs (AEDs) [4]. The goal of this treatment is to achieve total absence of seizures with minimal side effects but, for many patients, the best outcome is to achieve optimal seizure control with negligible adverse reactions [5]. Over the past 50 years, AED therapeutic monitoring has been used as a tool to manage the effects of these medications in patients with epilepsy [4]. Since 1989, eighteen novel AEDs have been developed, contributing to the increase in significance of the application of drug monitoring in the clinical management of epileptic patients [5]. Nowadays, 27 AEDs are licensed, making them among the most commonly monitored drugs via therapeutic drug monitoring (TDM) [4,5].

Blood/plasma samples are still commonly used for TDM of AEDs, but this type of sample presents limitations such as invasive collection, which can limit the number of collected samples, and requires specialized personnel, specific equipment, and facilities, allied with an increased amount of labor from the collection process to the testing step. For these reasons, there has been an attempt to create and evaluate new simple, easy, and viable non-invasive procedures, including methods based on the sampling of oral fluid [6,7,8].

Oral fluid is an alternative not only to blood, but also to urine, since it allows better surveillance during sample collection, which is faster and less invasive when compared to blood, reducing the risk of sample tampering [7]. It is becoming even more relevant since it reflects the free concentration of many AEDs in both serum and plasma while being easily collected several times without requiring experienced personnel, and thus can be more suitable for young and older patients. Also, the free drug fraction easily reflects the pharmacological effects of these medicines [9,10,11]. Nonetheless, oral fluid still presents several disadvantages. Its composition can change due to several factors such as age, gender, smoking habits, circadian rhythm, current health situation, or diet, or with the usage of a few medicines. Some of these factors can contribute to the contamination of the oral cavity, affecting the results of the drug analysis. As previously mentioned, oral fluid can be easily collected multiple times, but the obtained volume is still limited and it can contain small amounts of analyte; sensitive detection methods are thus required for this matrix analysis [12].

The samples are frequently not analyzed for weeks or even months after being collected, which leads to tampered results that are lower than would be expected immediately after collection [13]. Also, there is a huge chance that these values will not correlate with the quality control parameters, since analyte degradation can occur throughout this delay, affecting the detected analyte concentrations. Additionally, there are several problems with the storage and transportation of these samples, since the storage conditions, an important factor in maintaining the qualitative and quantitative drug characteristics in the samples, are most often undefined [13,14].

The use of dried saliva spots (DSSs) is a well-known alternative to the classic oral fluid collection techniques, requiring low sample volumes. In this technique, oral fluid is collected by spotting on a filter paper and is posteriorly eluted with an organic solvent in order to extract the analytes [15,16]. The fact that the samples are dried on a filter paper simplifies their transportation and storage, while ensuring the analytes’ excellent long-term stability. Also, this technique does not require high costs for the sample analysis and has been shown to be a promising tool for TDM and diagnosis of several congenital, metabolic, and hereditary disorders [16,17]. Recently, new DSS-based methods have been showcased, specially methods that associate DSS with GC-MS/MS [16,17,18,19], HPLC-DAD [20], LC-MS/MS [21,22], LC-MS [23], ELISA [24], modified competitive oligonucleotide priming-polymerase chain reaction (mCOP-PCR) [25], and SERS [26].

Previously, this research group developed and verified a new analytical technique utilizing DSS coupled with HPLC-DAD to determine carbamazepine (CBZ), carbamazepine-10,11-epoxide (CBZ-EP), phenytoin (PHT), and phenobarbital (PB) and, in this study, we apply the same method to assess the stability of the same AEDs [20]. Through a design of experiments (DOE), we evaluated the effects of the presence and absence of three different preservatives (sodium fluoride, sodium azide, and ascorbic acid) and of four different storage conditions for the DSS (time, temperature, preservative concentration, and presence or absence of light). Based on this DOE, this study was carried out for 63 days with the optimal storage conditions. To the best of our knowledge, this is the first study to combine DSS and HPLC-DAD to assess the stability of these antiepileptic drugs in oral fluid samples.

## 2. Results and Discussion

### 2.1. Optimisation of the Stability Protocol

This study aimed to evaluate and optimize the influence of several factors—namely light exposure, storage temperature, preservative concentration, and storage duration—on the stability of the analytes. By analyzing the absolute peak areas, we constructed Pareto charts, interaction plots, and main effects diagrams. The length of the bars in the Pareto charts identifies the variables with the most significant influence on analyte stability, whereas the main effects plots illustrate how the response changes according to the levels of each tested parameter (e.g., refrigeration versus room temperature). All effects were assessed using the signal obtained through HPLC-DAD analysis.

**Figure 1:**
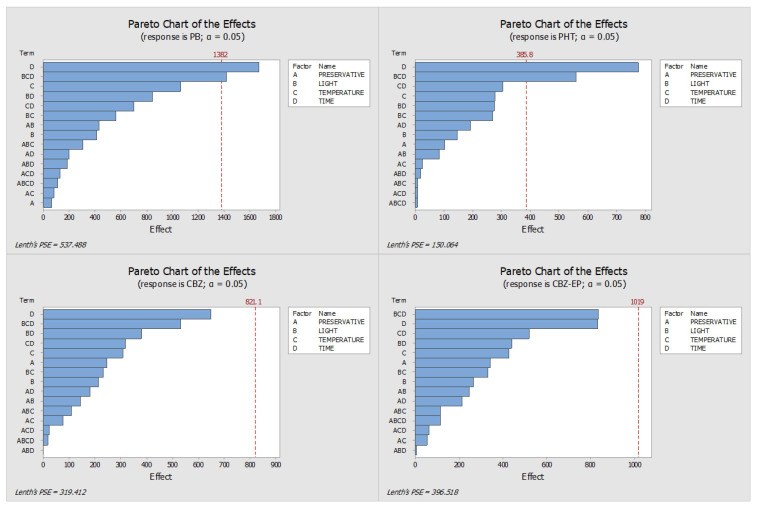
Pareto charts derived from DOE analysis for samples preserved with ascorbic acid. (PHT—phenytoin, PB—phenobarbital, CBZ—carbamazepine, CBZ-EP—carbamazepine-10,11-epoxide). The impact of each condition—A: preservative concentration, B: light exposure, C: storage temperature, and D: storage time—is represented by the length of the bars.

**Figure 2:**
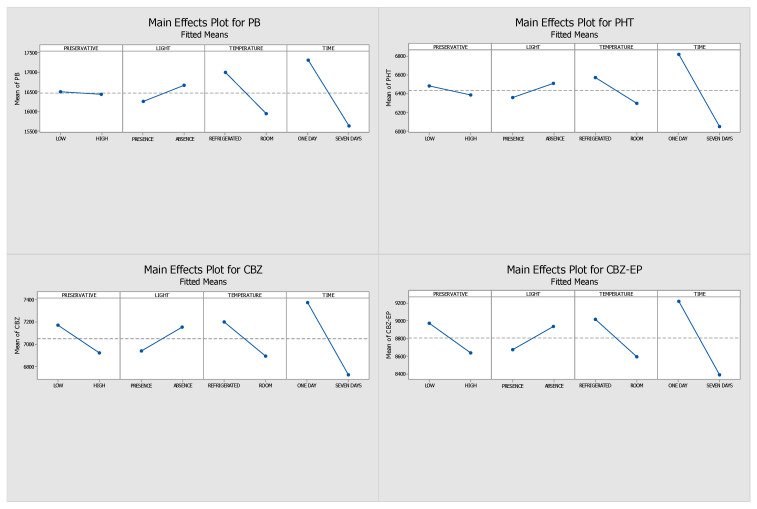
Main effects plots generated through DOE for samples preserved with ascorbic acid. (PHT—phenytoin, PB—phenobarbital, CBZ—carbamazepine, CBZ-EP—carbamazepine-10,11-epoxide). The influence of each factor—preservative concentration (low vs. high), light exposure (present vs. absent), temperature (4 °C vs. 25 °C), and storage duration (1 vs. 7 days)—is indicated by the slope of each line.

**Figure 3:**
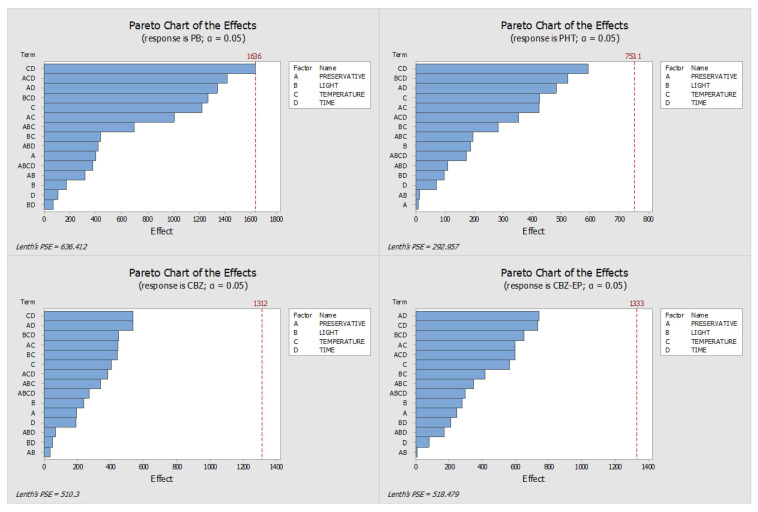
Pareto charts generated using DOE for samples in which sodium azide was used as the preservative (PHT—phenytoin, PB—phenobarbital, CBZ—carbamazepine, CBZ-EP—carbamazepine-10,11-epoxide). The influence of each condition—A: preservative concentration, B: light exposure, C: storage temperature, and D: storage time—is represented by the length of the bars.

**Figure 4:**
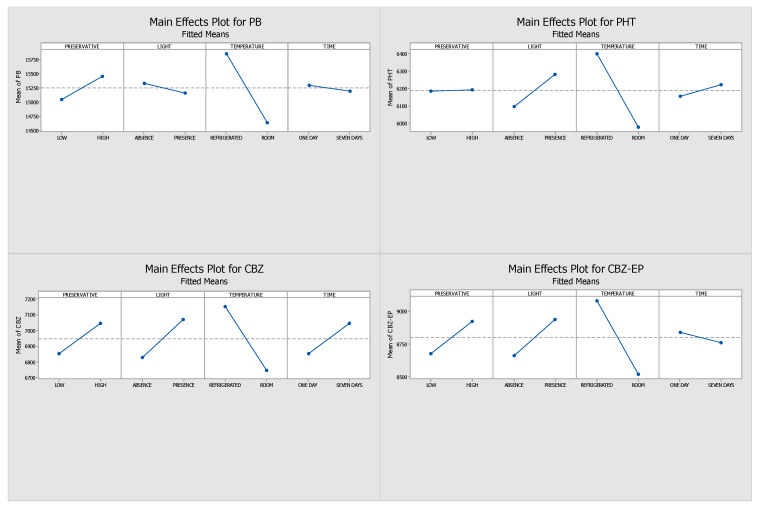
Main effects plots generated through DOE for samples preserved with sodium azide. (PHT—phenytoin, PB—phenobarbital, CBZ—carbamazepine, CBZ-EP—carbamazepine-10,11-epoxide). The influence of each tested parameter—preservative concentration (low vs. high), light exposure (presence vs. absence), storage temperature (4 °C vs. 25 °C), and storage duration (1 vs. 7 days)—is reflected in the slope of the lines.

**Figure 5:**
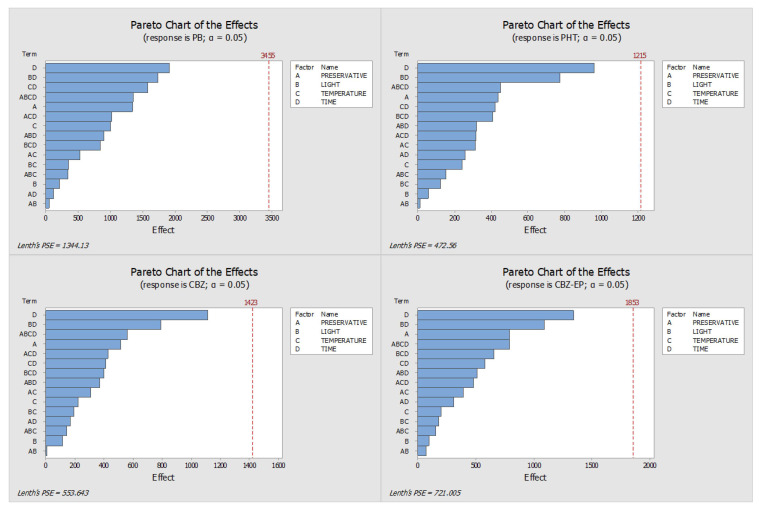
Pareto charts obtained with the DOE when sodium fluoride was used as preservative (PHT—phenytoin, PB—phenobarbital, CBZ—carbamazepine, CBZ-EP—carbamazepine-10,11-epoxide). The effects of the conditions (A—Preservative, B—Light, C—Temperature, and D—Time) are highlighted by the length of the bars.

**Figure 6:**
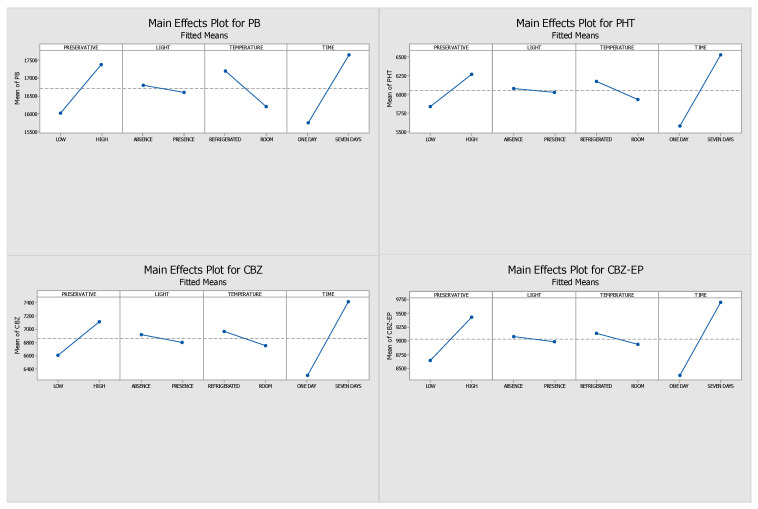
Main effects plots generated through DOE for samples preserved with sodium fluoride. (PHT—phenytoin, PB—phenobarbital, CBZ—carbamazepine, CBZ-EP—carbamazepine-10,11-epoxide). The influence of each tested factor—preservative concentration (low vs. high), light exposure (present vs. absent), temperature (4 °C vs. 25 °C), and storage time (1 vs. 7 days)—is indicated by the slope of the lines.

Figure 1 and Figure 2 display the Pareto and main effects plots, respectively, for all four analytes when ascorbic acid was used as the preservative. Similarly, Figure 3 and Figure 4 present the corresponding graphs for each target analyte in the presence of sodium azide as the preservative. Lastly, Figure 5 and Figure 6 show the same two graphs, but the responses were obtained when samples were preserved with sodium fluoride.

An evaluation of the Pareto charts based on the absolute peak areas of the analytes (Figure 1) indicates that the stability of PB and PHT was notably influenced by storage time, along with the combined effect of light exposure, temperature, and duration of storage when ascorbic acid was used as a preservative. For CBZ, storage time emerged as the main factor influencing stability, although no individual condition showed a statistically significant impact. The results for CBZ-EP followed a similar pattern to those observed for CBZ.

The main effects plots provide insight into the optimal storage conditions for the analytes under study. For the samples with ascorbic acid as a preservative, the main effect plots (Figure 2) exhibited similar responses across the four conditions for all analytes. Optimal results were obtained on day one of storage under conditions that included a low preservative concentration, protection from light, and refrigeration at 4 °C. Ultimately, since a greater response strongly suggests a higher stability, it is reasonable to draw the conclusion that the four highlighted conditions—300 ng/mL concentration of ascorbic acid, short storage time, absence of light, and refrigeration at 4 °C—were the ones in which all four analytes demonstrated higher stability.

No individual factor or combination of factors showed a statistically significant effect on the stability of any of the four analytes when sodium azide was used as a preservative (Figure 3). However, the combination of storage temperature and duration approached the significance threshold for PB, suggesting a near-significant impact. For PHT, this same factor combination exhibited a noticeable effect, while both this interaction and the combination of preservative concentration and storage time had a comparable influence on CBZ. In the case of CBZ-EP, the interaction between storage time and preservative concentration had a slightly greater impact than the temperature–time combination.

As shown in the main effects plots (Figure 4), all analytes demonstrated better responses when lower concentrations of sodium azide were used, aligning with the trends observed for ascorbic acid. Additionally, refrigerated conditions (4 °C) resulted in better stability across all compounds. Notably, PHT, CBZ, and CBZ-EP showed enhanced responses when stored in the presence of light, whereas PB exhibited a greater response in the absence of light.

In terms of storage duration, PB and CBZ-EP showed the strongest responses on the first day, while for PHT and CBZ, the opposite trend was observed, with responses improving over time. Overall, for sodium azide, the optimal conditions included low preservative concentration, refrigeration, and light exposure, and storage temperature emerged as the most influential factor affecting analyte stability.

Finally, oral fluid samples preserved with sodium fluoride were exposed to the same experimental conditions. Although storage time emerged as the most influential factor in the Pareto charts, no individual variable showed a statistically significant impact on the stability of the analytes (Figure 5).

As illustrated in the main effects plots (Figure 6), preservative concentration and storage duration were the most influential factors affecting sample stability. All four analytes exhibited enhanced stability by day seven when stored with a high concentration of sodium fluoride. In general, the samples showed consistent behavior under all tested conditions, with improved responses observed under refrigerated conditions (4 °C) and in the absence of light. These findings suggest that, for sodium fluoride, optimal storage involves using a high preservative concentration, maintaining low temperatures, and protecting samples from light exposure.

Based on the main effects plots (Figure 6), it is possible to infer that both preservative concentration and storage duration had the biggest effects on the stability of the analytes. On the seventh day, all four analytes showed improved stability when a high sodium fluoride concentration was used. It is possible to say that the results were generally consistent throughout the four conditions, with the analytes exhibiting a stronger response when the samples were kept in the dark at 4 °C. For sodium fluoride, the ideal storage conditions proved to be light-free, having a high preservative content, and refrigerated.

### 2.2. Stability Study Without Preservative

Following the stability assessment of analytes in the presence of preservatives, a new DOE was carried out using samples without any preservative. In this phase, oral fluid was fortified with the same four AEDs and applied to DSS cards, following the same procedure as in the previous experiments, but omitting the addition of preservatives. The objective was to investigate how the samples behaved under identical conditions (storage duration, temperature, and light exposure) in the absence of a preservative. These results will subsequently be compared with those obtained from samples containing preservatives.

**Figure 7:**
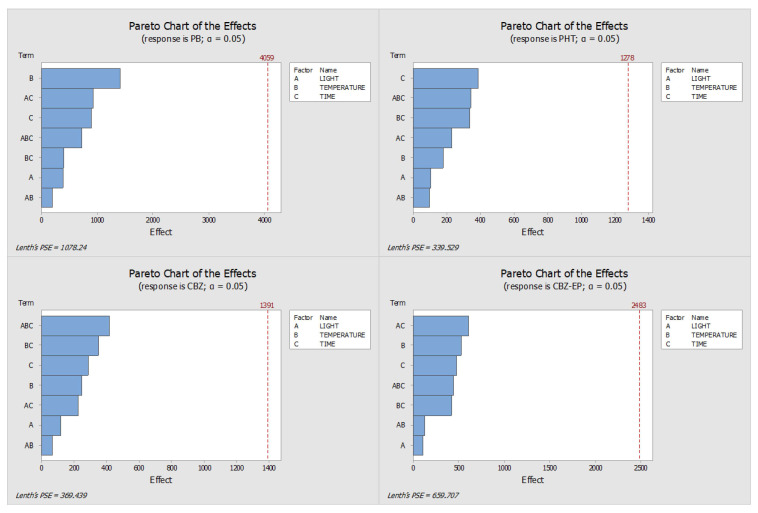
Pareto charts obtained with the DOE when no preservative was used. (PHT—phenytoin, PB—phenobarbital, CBZ—carbamazepine, CBZ-EP—carbamazepine-10,11-epoxide). The effects of the conditions (A—Light, B—Temperature, and C—Time) are highlighted by the length of the bars.

**Figure 8:**
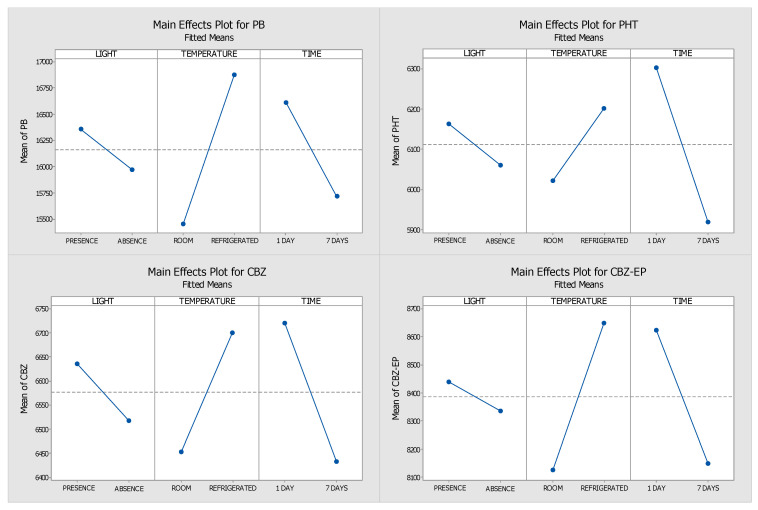
Main effects plots generated through DOE for samples without preservative. (PHT—phenytoin, PB—phenobarbital, CBZ—carbamazepine, CBZ-EP—carbamazepine-10,11-epoxide). The influence of the tested parameters—light exposure (present vs. absent), temperature (4 °C vs. 25 °C), and storage duration (1 vs. 7 days)—is indicated by the slope of the lines.

As in the preservative samples, the impact of these factors was assessed using the analytes’ absolute peak areas. The resulting Pareto and main effects graphs are shown in Figure 7 and Figure 8, respectively.

The Pareto charts presented in Figure 7 did not show any statistically significant effects of the tested variables on analyte stability. However, storage temperature, storage duration, light exposure, and the interaction between light exposure and storage time emerged as the most influential factors across all four analytes.

The reactions of all four analytes to the three conditions were comparable, with the first day showing stronger responses when both light and refrigeration were present (Figure 8). The two most important variables affecting the stability of these analytes were temperature and storage duration. For PB and CBZ-EP, temperature had a stronger effect than storage duration, whereas PHT and CBZ revealed the opposite results. Based on the DOE and the interpretation of the graphs, it is possible to infer that the optimal storage conditions are refrigeration at 4 °C in the presence of light.

### 2.3. Final Conditions for the Long-Term Stability Assay

To determine the optimal storage conditions for the DSS, the findings from all Pareto and main effects analyses were integrated, taking into account the results from both sets of samples—those with added preservative and those without. It is clear from the analysis of the Pareto charts for all three preservatives (Figure 1, Figure 3 and Figure 5) that, for the ascorbic acid samples, there were two factors with a substantial impact on the stability of PB and PHT, although neither CBZ nor CBZ-EP exhibited any significant effects.

On the seventh day of analysis, the ascorbic acid samples showcased greater absolute peak areas than the sodium azide and sodium fluoride samples, indicating a reduced analyte loss. These results led to the selection of ascorbic acid as the preservative. Based on their greater and consistent results, low preservative concentration, 4 °C storage, and no light were selected as the best conditions for the long-term stability assay.

To complement these findings, new DSS samples were prepared in triplicate using both ascorbic acid and no preservative, and stored for seven days. For the preservative set, a low concentration of ascorbic acid, protection from light, and refrigeration were applied, whereas the non-preservative set was stored under light exposure and refrigerated conditions. The response to these conditions was evaluated and is presented in Table 1.

The results in Table 1 show minor differences between the responses of the analytes in both conditions after 7 days of storage. While variations are observed, particularly for CBZ, they are not substantial, suggesting that the impact of the preservative within this timeframe may be limited.

### 2.4. Long-Term Sample Stability

A study comprising nine assays was carried out over a period of sixty-three days, in order to assess the long-term stability of the four AEDs. The stability assays were performed on the following days: 1 (sample preparation day), 7, 14, 21, 28, 35, 42, 49, 56, and 63. In addition, samples without preservatives were prepared and analyzed on the same days to allow comparison of the preservative’s effect on the stability of the selected compounds.

Analyte concentrations of 10 µg/mL and 0.5 µg/mL were used to create both preservative and non-preservative samples, which were then held for 63 days under the DOE-optimized storage conditions. Duplicate samples were made for each of the nine assays, and, on day one, the concentration for each analyte was set at 100%. Analyte instability was defined as a variation greater than 20% from the initial analyte concentration. Although standard guidelines typically recommend a maximum deviation of ±15%, some authors accept a threshold of ±20% due to the inherent variability of dried matrix spot samples [27].

Regarding the results presented in Table 2 and Table 3, PB at a concentration of 10 µg/mL exhibited a marked decrease after 35 days in samples containing preservative. In contrast, at 0.5 µg/mL, the compound remained stable up to day 21. When compared to the samples without preservative, PB at 10 µg/mL remained stable for an additional 14 days, while at 0.5 µg/mL, better stability was observed in the absence of preservative, with stability maintained until day 28.

For PHT at 10 µg/mL, the analyte was stable up to day 35 in the presence of preservative, whereas a significant decline was observed after day 21 in the non-preserved samples. At 0.5 µg/mL, PHT followed a similar trend to that of PB, with non-preserved samples showing extended stability (35 days) compared to those with preservative (28 days).

CBZ demonstrated noticeable degradation after days 35 and 21 in preserved samples at concentrations of 10 µg/mL and 0.5 µg/mL, respectively. By comparison, non-preserved samples at 10 µg/mL remained stable for 14 days, while those at 0.5 µg/mL showed stability up to day 28.

Finally, CBZ-EP remained stable for 28 days in samples at 10 µg/mL with preservative, while in the absence of preservative, stability was maintained for 14 days. At the lower concentration of 0.5 µg/mL, samples without preservative remained stable up to 28 days, whereas those containing preservative showed stability for approximately 21 days.

In the study conducted by Carvalho et al. [20], the same four analytes were shown to be stable for 21 days when stored on DSS cards at room temperature under benchtop conditions. Samples were prepared in triplicate at three quality control (QC) levels (0.2, 1.0, and 6.0 µg/mL), and the stability findings were consistent across all tested concentrations.

## 3. Materials and Methods

### 3.1. Reagents and Standards

Acetonitrile (ACN; Prolabo, Lisbon, Portugal), triethylamine (TEA; Merck Co., Darmstadt, Germany), isopropanol (Fisher Chemical, Loughborough, UK), glacial acetic acid (Sigma-Aldrich, Sintra, Portugal), and methanol (Merck Co., Darmstadt, Germany) were all of pro-analysis grade. Ketoprofen (KTP), used as the internal standard (IS), was obtained from Sigma-Aldrich (Sintra, Portugal), as were the analytical standards of PHT, PB, CBZ, and CBZ-EP. Whatman™ 903 protein saver cards were also sourced from Sigma-Aldrich (Sintra, Portugal). Deionized water was produced using a Milli-Q purification system (Millipore, Billerica, MA, USA).

Standard solutions of PHT, PB, CBZ, and CBZ-EP at concentrations of 200, 20, and 2 μg/mL were prepared by sequential dilution of stock solutions in methanol. A working solution of the internal standard (KTP) was also prepared in methanol at a final concentration of 5 μg/mL. All prepared solutions were stored at 4 °C and protected from light.

### 3.2. Biological Specimens

For this study, drug-free oral fluid was collected from at least six laboratory staff volunteers using the unstimulated spitting method, following a minimum one-hour fasting period to reduce variability and potential contamination. The individual samples were pooled to obtain a homogeneous matrix and then stored at –20 °C until analysis. Before application to the dried saliva spot (DSS) cards, the pooled oral fluid was thawed and centrifuged at 3500 rpm for 15 min to remove debris and ensure sample clarity.

### 3.3. HPLC-DAD Conditions

Chromatographic analysis was performed according to the method described by Carvalho et al. [20]. An Agilent 1290 Infinity HPLC system equipped with a quaternary pump and coupled to a 1290 Infinity diode array detector (G4212A DAD) (Soquímica, Lisbon, Portugal) was used. Separation of the AEDs and internal standard (IS) was achieved using a Zorbax Eclipse Plus C18 analytical column (5 μm, 4.6 × 250 mm i.d.) from Agilent Technologies (Soquímica, Lisbon, Portugal).

The HPLC–DAD operated in isocratic mode with a binary mobile phase. Solution A, comprising 100% acetonitrile, accounted for 20% of the mobile phase, while Solution B consisted of water:methanol:triethylamine (75.5:24.2:0.3, *v*/*v*) adjusted to pH 6.5, making up the remaining 80%. The total runtime was 20 min, with a flow rate of 0.8 mL/min. The autosampler was maintained at 4 °C, the column temperature was set to 35 °C, and the injection volume was 50 μL. Detection of the analytes was carried out at 210 nm, a wavelength commonly used for the analysis of these compounds in the literature [28,29,30,31,32,33,34].

### 3.4. Sample Preparation

The preparation of oral fluid samples followed the study established by Carvalho et al. [20]. Following thawing at room temperature, oral fluid samples were centrifuged at 3500 rpm for 15 min. Subsequently, 50 μL of each sample was applied to a dried saliva spot (DSS) card, allowed to dry for one hour, and then excised around the marked sampling area before being transferred to a glass tube.

The sample was eluted using 1 mL of acidified methanol (acidified with glacial acetic acid to attain a pH of 5.5) and 20 μL of the internal standard (IS) working solution, in order to carry out the liquid extraction. After 5 min of agitation at 70 rpm on a roller mixer, the organic phase was moved into a fresh clean glass tube. Then, for 15 min, the new glass tube was centrifuged at 3500 rpm. The extract was then evaporated to dryness under a gentle stream of nitrogen, and the residue was reconstituted in 80 μL of mobile phase prior to injection into the HPLC–DAD system.

The analytical method had been previously validated with respect to selectivity, linearity, limits of detection and quantification, precision, accuracy, and recovery, in accordance with international bioanalytical validation guidelines [20]. Linearity was established between 0.1 and 10 μg/mL for all analytes, with determination coefficients consistently exceeding 0.998. The limit of detection was 0.05 μg/mL, and the lower limit of quantification was 0.1 μg/mL. Intra-day and inter-day precision values were all below 13%, with relative errors within ±15%. Intermediate precision, evaluated over five days at three quality control levels, also demonstrated CVs below 13% and accuracy within ±5%. Recovery values ranged from 43% to 57%, depending on the analyte under study.

### 3.5. Design of Experiments

Design of experiments (DOE) is a ground-breaking technique that maximizes the experimental parameters while promoting the understanding of how these factors, either alone or in combination, can impact the experimental outcomes [35,36]. Employing DOE enables the collection of extensive experimental data with optimized cost and time. Additionally, it has been widely used in a several industries including the food, engineering, environment, energy, pharmaceutical, and biological sectors [37].

The present study aimed to optimize the long-term stability of the DSS cards analytes by conducting a DOE study to evaluate the compounds’ response to four conditions: storage time (1 day and 7 days), preservative concentration (300 ng/mL and 600 ng/mL for ascorbic acid, 1% and 2% for sodium fluoride, and 0.1% and 0.2% for sodium azide), absence or presence of light, and storage temperature (4 °C and 25 °C). The preservatives were chosen based on the literature [16] and specific norms were followed for each of the three preservative concentrations. The manufacturer’s guidelines were followed for sodium azide concentrations, and the International Association of Forensic Toxicologists (TIAFT)—Committee on Systematic Toxicological Analysis sample guidelines were followed for sodium fluoride concentrations [38]. Lastly, the ascorbic acid concentrations were based on the study by Nielsen et al. [39]. To determine whether the outcomes of the best preservative circumstances were noticeably superior than the outcomes of the no-preservative settings, a second DOE assay was conducted to evaluate how the samples without a preservative responded to the other three previously mentioned parameters.

In Table 4 it is possible to observe the experimental design matrix used to evaluate the previously listed factors at two levels (2^4^), while in Table 5 presents the experimental design matrix used to evaluate the three factors applied to samples without preservative at two levels (2^3^).

### 3.6. Long-Term Stability Evaluation

In order to assess the long-term stability, the DSS-spiked samples were produced in duplicate and held for 1, 7, 14, 28, 35, 42, 49, 56, and 63 days under the ideal storage conditions found in the DOE research. The response was assessed by converting the measured relative peak areas to concentration through a newly created calibration curve. Additionally, it was compared with the results of fresh DSS samples. The newer samples were dried for 24 h, which was the minimum time interval for the long-term stability assay. For day 1, the target analyte concentration was established as 100%.

## 4. Conclusions

This study evaluated the stability of PB, PHT, CBZ, and CBZ-EP in oral fluid samples stored as DSS samples, with and without the addition of preservatives (sodium azide, sodium fluoride, and ascorbic acid), under several storage conditions. A DOE approach was employed to optimize the parameters affecting analyte stability. The optimal storage conditions were identified as refrigeration at 4 °C, absence from light, and the use of a low concentration of ascorbic acid (300 ng/mL).

Under these conditions, all four AEDs at 10 µg/mL remained stable for over 28 days. At a lower concentration (0.5 µg/mL), samples stored without preservative consistently showed better stability than those with preservative. Specifically, AEDs remained stable for an additional 7 days.

This is the first study to assess the effect of different storage conditions on the stability of these four AEDs in oral fluid spotted onto a DSS. The findings highlight that while ascorbic acid at 300 ng/mL is effective for maintaining stability at higher concentrations, preservative-free storage may be more suitable for lower analyte levels. Overall, this work provides valuable evidence supporting the use of DSSs as a reliable tool for the long-term monitoring of AEDs in TDM.

## Figures and Tables

**Table 1:** Mean responses of the analytes in newly prepared samples stored at 4 °C under light exposure with ascorbic acid (A), and mean responses of the analytes in samples without ascorbic acid stored at 4 °C in the absence of light (B). A low concentration of ascorbic acid was used for the preservative condition.

	1st Day		7th Day	
Analyte	A	B	A	B
PB	19,324	19,814	18,747	19,610
PHT	7553	7801	7277	7344
CBZ	8002	8027	7659	6636
CBZ-EP	9806	10,190	9344	9672

CBZ: carbamazepine; CBZ-EP: carbamazepine-10,11-epoxide; PB: phenobarbital; PHT: phenytoin.

**Table 2:** Stability results for the antiepileptic drugs PB, PHT, CBZ, and CBZ-EP over a 63-day storage period (*n* = 2) at a concentration of 10 µg/mL.

		With Ascorbic Acid		No Preservative	
Analyte	Days	Relative Loss (%)	CV (%)	Relative Loss (%)	CV (%)
PB	1	0	5.3	0	1.3
	7	11	0.9	−8	5.8
	14	3	1.9	−16	1.2
	21	−2	4.3	−21	10.6
	28	−9	2.8	−26	2.6
	35	−29	0.1	−34	1.9
	42	−30	2.1	−39	7.3
	49	−36	1.9	−45	8.3
	56	−43	5.7	−51	2.3
	63	−49	10.1	−55	5.2
PHT	1	0	2.6	0	5.7
	7	14	2.3	−9	2.6
	14	5	8.2	−17	1.1
	21	−1	9.3	−22	1.2
	28	−9	5.7	−29	9.3
	35	−24	4.5	−35	7.4
	42	−37	2.9	−38	12.9
	49	−42	6.0	−48	7.1
	56	−46	4.0	−54	8.2
	63	−51	6.6	−60	5.2
CBZ	1	0	4.8	0	1.1
	7	13	0.4	−7	4.6
	14	1	1.6	−19	1.3
	21	−4	8.7	−24	3.8
	28	−13	4.3	−31	1.1
	35	−27	5.4	−37	9.6
	42	−34	10.2	−42	1.8
	49	−35	7.8	−48	6.3
	56	−45	0.5	−53	2.2
	63	−47	0.1	−58	3.6
CBZ-EP	1	0	1.0	0	1.3
	7	15	1.1	−8	1.5
	14	6	0.5	−18	5.3
	21	−5	2.6	−22	3.9
	28	−11	0.4	−30	7.6
	35	−29	0.3	−36	7.8
	42	−35	0.7	−41	0.8
	49	−39	9.5	−45	4.5
	56	−41	1.1	−53	2.5
	63	−45	1.3	−57	5.2

CBZ: carbamazepine; CBZ-EP: carbamazepine-10,11-epoxide; PB: phenobarbital; PHT: phenytoin.

**Table 3:** Results of the stability assay of the antiepileptic drugs PB, PHT, CBZ and CBZ-EP through 63 days of storage (*n* = 2) at 0.5 µg/mL.

		With Ascorbic Acid		No Preservative	
Analyte	Days	Relative Loss (%)	CV (%)	Relative Loss (%)	CV (%)
PB	1	0	5.4	0	2.6
	7	−8	1.8	6	1.1
	14	−17	5.2	10	8.1
	21	−20	6.7	−1	4.7
	28	−23	1.2	−11	8.9
	35	−38	3.7	−24	5.6
	42	−45	8.8	−33	3.5
	49	−66	4.8	−43	1.7
	56	−69	3.4	−55	2.2
	63	−64	8.1	−69	3.1
PHT	1	0	4.3	0	4.7
	7	−10	1.6	7	9.5
	14	−16	4.9	11	6.1
	21	−19	2.2	−2	7.2
	28	−22	2.6	−10	2.6
	35	−34	3.5	−22	1.7
	42	−44	9.2	−34	5.1
	49	−69	8.4	−44	1.3
	56	−71	5.6	−53	2.1
	63	−60	1.0	−65	4.2
CBZ	1	0	7.8	0	5.9
	7	−10	3.6	8	4.9
	14	−19	0.6	13	1.6
	21	−20	1.3	−1	8.8
	28	−23	3.2	−12	6.5
	35	−36	2.4	−24	3.7
	42	−49	1.6	−36	0.5
	49	−67	0.9	−47	4.4
	56	−73	8.7	−58	5.2
	63	−62	6.7	−67	5.6
CBZ-EP	1	0	8.5	0	1.3
	7	−9	7.9	8	7.7
	14	−15	6.4	14	2.9
	21	−18	4.3	2	6.5
	28	−21	4.1	−9	5.3
	35	−39	8.0	−21	2.1
	42	−46	10.0	−32	9.2
	49	−64	10.1	−44	10.4
	56	−68	8.4	−55	5.1
	63	−60	3.2	−68	3.4

CBZ: carbamazepine; CBZ-EP: carbamazepine-10,11-epoxide; PB: phenobarbital; PHT: phenytoin.

**Table 4:** Experimental design matrix (2^4^ factorial design) with four factors at two levels, applied to samples containing preservative.

Run Order	Preservative Concentration	Time	Temperature	Light
1	Low	One Day	4 °C	Absence
2	High	One Day	4 °C	Absence
3	Low	One Day	4 °C	Presence
4	High	One Day	4 °C	Presence
5	Low	One Day	25 °C	Absence
6	High	One Day	25 °C	Absence
7	Low	One Day	25 °C	Presence
8	High	One Day	25 °C	Presence
9	Low	Seven Days	4 °C	Absence
10	High	Seven Days	4 °C	Absence
11	Low	Seven Days	4 °C	Presence
12	High	Seven Days	4 °C	Presence
13	Low	Seven Days	25 °C	Absence
14	High	Seven Days	25 °C	Absence
15	Low	Seven Days	25 °C	Presence
16	High	Seven Days	25 °C	Presence

Each line of the matrix represents an assay.

**Table 5:** Experimental design matrix (2^3^ factorial design) with three factors at two levels, applied to samples without preservative.

Run Order	Time	Temperature	Light
1	1 Day	25 °C	Absence
2	1 Day	25 °C	Presence
3	1 Day	4 °C	Absence
4	1 Day	4 °C	Presence
5	7 Days	25 °C	Absence
6	7 Days	25 °C	Presence
7	7 Days	4 °C	Absence
8	7 Days	4 °C	Presence

Each line of the matrix represents an assay.

## Data Availability

The data presented in this study are available on request from the corresponding authors.

## Abbreviations

The following abbreviations are used in this manuscript:

AEDs	Antiepileptic drugs
TDM	Therapeutic drug monitoring
DSS	Dried saliva spots
GC-MS/MS	Gas chromatography–tandem mass spectrometry
HPLC-DAD	High-performance liquid chromatography with diode array detection
LC-MS/MS	Liquid chromatography–tandem mass spectrometry
LC-MS	Liquid chromatography–mass spectrometry
ELISA	Enzyme-linked immunoassay
mCOP-PCR	Modified competitive oligonucleotide priming-polymerase chain reaction
SERS	Surface-enhanced Raman scattering
CBZ	Carbamazepine
CBZ-EP	Carbamazepine-10,11-epoxide
PHT	Phenytoin
PB	Phenobarbital
DOE	Design of experiments
